# COVID-19 Vaccination Rates and Predictors of Uptake Among Adults With Coronary Heart Disease: Insight From the 2022 National Health Interview Survey

**DOI:** 10.7759/cureus.52480

**Published:** 2024-01-18

**Authors:** Victor C Ezeamii, Victor C Ofochukwu, Charity Iheagwara, Tracy Asibu, Oluwatoyin Ayo-Farai, Yonas H Gebeyehu, Eunice O Kaglo, Moses C Odoeke, Olaoluwa M Adeyemi, Hameed O Shittu, Okelue E Okobi

**Affiliations:** 1 Public Health, Jiann-Ping Hsu College of Public Health, Georgia Southern University, Statesboro, USA; 2 Medicine, Ebonyi State University, Abakaliki, NGA; 3 Medicine and Surgery, Hospital Corporation of America (HCA) Hospital Pearland, Pearland, USA; 4 Infectious Diseases, Saint Michael's Medical Center, Newark, USA; 5 Biomedical Informatics, University of Texas Health Science Center at Houston, Houston, USA; 6 Epidemiology and Public Health, Jiann-Ping Hsu College of Public Health, Georgia Southern University, Statesboro, USA; 7 Medicine, Addis Ababa University, Addis Ababa, ETH; 8 General Practice, Southwestern University School of Medicine, Calgary, CAN; 9 Internal Medicine, Al Ruwaydah General Hospital, Riyadh, SAU; 10 Internal Medicine, Afe Babalola University, Ado, NGA; 11 Internal Medicine/Nephrology, William Osler Health System - Etobicoke General Hospital, Toronto, CAN; 12 Internal Medicine, Richmond Gabriel University, Kingstown, VCT; 13 Internal Medicine, Federal Medical Centre, Abeokuta, NGA; 14 Family Medicine, Larkin Community Hospital Palm Springs Campus, Miami, USA; 15 Family Medicine, Medficient Health Systems, Laurel, USA; 16 Family Medicine, Lakeside Medical Center, Belle Glade, USA

**Keywords:** older age, covid-19, prevention, vaccine, coronary heart disease

## Abstract

Introduction: COVID-19 has become a burden to all nations across the globe, and vaccination currently remains the most effective means of fighting the SARS-COV-2 pandemic. From the time of approval and subsequent distribution of the various COVID-19 vaccines, nearly 72.3% (5.5 billion) of the globe's population have been vaccinated, leaving slightly more than a quarter of the globe's population at risk. With the approval and availability of booster vaccine dosages to individuals with chronic conditions, including coronary heart disease (CHD), it is vital to comprehend the factors underlying the uptake of COVID-19 vaccination in such subgroups. Further, the American Heart Association recommends vaccination against COVID-19 in populations with coronary heart disease (CHD). This is because they are more likely to experience severe outcomes due to COVID-19 infection. This study assesses the uptake of COVID-19 vaccines as well as predictors of its uptake.

Methods: Using the 2022 survey data from the National Health Interview Survey (NHIS), 1,708 adults ≥ 40 years with CHD who responded yes/no to whether they had received the vaccine were identified. A Pearson's chi-square test was used to ascertain differences among those who had received the vaccine and those who had not. A logistic regression (multivariate regression) was used to evaluate predictors of COVID-19 vaccination.

Results: About 1,491/1,708 (86.8%) adults ≥ 40 years reported being vaccinated against COVID-19. Among them, 1,065/1,491 (68.4%) had received more than two vaccination doses. The predictors of COVID-19 vaccination were older age (odds ratio (OR): 2.01 (95% confidence interval (CI): 1.40-2.89), p < 0.001), ratio of family income to poverty threshold of 1 and above (OR: 2.40 (95% CI: 1.58-3.64), p < 0.001), having a college degree (OR: 3.09 (95% CI: 1.85-5.14), p < 0.001), and being insured (OR: 3.26 (95% CI: 1.03-10.26), p = 0.044).

Conclusion: The findings of the study have indicated that 68.4% of adults 40 years and above with CHD have been vaccinated against COVID-19 and have received more than two doses of vaccines. More than half have followed recommendations and have received booster doses of the vaccine. Old age (above 40 years) and a higher socioeconomic class are associated with being more likely to follow COVID-19 vaccination guidelines. Despite the higher vaccination rate of 68.4% in the adults with heart diseases group, strategies for improving booster vaccine awareness alongside accessibility are needed to enhance additional dosage uptake, protect them against novel COVID-19 variants, and ensure the development of sustained immunity.

## Introduction

At the peak of the COVID-19 pandemic, the United States (US) ranked first in total pandemic deaths, contributing 19% of the global total, with the 12th worst COVID-19 cumulative mortality rates of all countries [1]. This was driven largely by COVID-19 being the third leading cause of death (697.5 deaths/million) only behind heart disease (1,287.7 deaths/million) and cancer (1,219.8 deaths/million) in the US [2]. Deaths in populations with cardiovascular diseases increased erratically in different regions of the country during this period, with higher mortality rates reported specifically in those with ischemic heart disease [3].

To address this problem, the US Food and Drug Administration approved the first COVID-19 vaccine for individuals 16 years of age and older in 2021 [4]. This was followed by further recommendations for booster doses in efforts to reduce the worrying trends in morbidity and mortality nationwide. Despite these recommendations, vulnerable groups have reportedly been shown to exhibit vaccine hesitancy, especially those in racial/ethnic minority groups, those who live in less densely populated regions, and some who work in healthcare [5]. Among the notable discernible trends in such vulnerable groups that may explain the observed vaccine hesitancy include the view that such groups have limited knowledge about COVID-19 and the various vaccines and their effectiveness. As such, the vulnerable groups are likely to believe that the vaccines are unsafe in addition to believing the COVID-19 myths. Averagely, the groups are less educated, are rural residents compared to individuals who believe that vaccines are safe, and have low incomes [5]. In a cross-sectional study published in 2022, COVID-19 vaccine uptake in the district of Columbia was reportedly low in residents with chronic medical conditions, with socioeconomic factors playing a key role in vaccination hesitancy [6]. In a systematic review of studies about COVID-19 vaccination in patients with diabetes mellitus, vaccine hesitation rates of about 30% were reported, with a lack of information mentioned as a significant reason for this finding [7]. Promising uptake in vaccination has however been reported in populations with cancer, with age, multiple comorbidities, and level of education identified as factors driving it [8].

Among populations with coronary heart disease, studies on COVID-19 vaccination uptake are insufficient. In this study, we assessed the uptake of COVID-19 vaccines among adults aged 40 years and above and with coronary heart disease, in addition to assessing the predictors of COVID-19 vaccine uptake. This study is important because COVID-19 vaccines have been found to be effective in preventing myocardial infarction and cerebrovascular events, particularly in long COVID populations with cardiovascular diseases [9,10].

## Materials and methods

Data

The 2022 National Health Interview Survey (NHIS) data was used for this study. Briefly, the NHIS is the principal source of information on the health of the civilian noninstitutionalized population of the US, with the main objective of monitoring the health of the population through the collection and analysis of data on a broad range of health topics. It is conducted continuously throughout the year by the National Center for Health Statistics (NCHS). Interviews are typically conducted in respondents' homes, but follow-ups to complete interviews may be conducted over the telephone. Further information about this NHIS data is available online [11].

Study design

This is a cross-sectional study utilizing the sample adult data from the 2022 National Health Interview Survey (NHIS). Since the data was publicly available, an institutional review board (IRB) review was not required.

Study population

**Figure 1:**
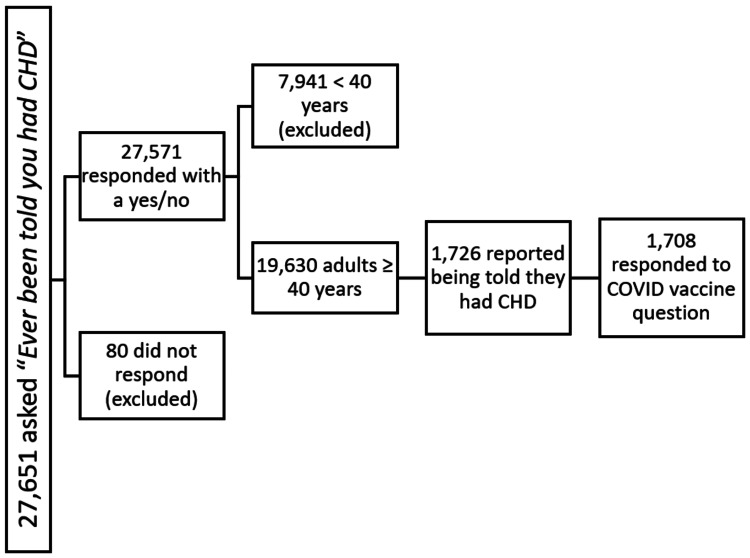
Sample identification for the study CHD: coronary heart disease, COVID: coronavirus disease

The data contained 27,651 respondents who were asked the question "Ever been told you had coronary heart disease (CHD)." From this, 27,571 responded yes or no to the interview question and 80 did not respond to the question. These nonresponders, making up 0.2% of the population, were excluded from the study. Among those who responded with a yes/no, 7,941 were under the age of 40 and were also excluded since CHD is rarely reported in this age group [12], leaving a population of 19,630. Among them, 1,726/19,630 representing approximately 13,000,000 US adults reported having CHD, of which only 1,708/1,726 responded to the question about whether they were vaccinated against COVID-19 (Figure 1).

Sociodemographic variables

The sociodemographic variables studied included age (40-64 and ≥65 years), ethnicity (non-Hispanic (NH) white, Hispanic, NH black, and NH others), gender (female and male), level of education (graduate from college and did not graduate from college), family income poverty ratio (<1 and ≥1), and insurance status (uninsured and insured).

Comorbidities

Respondents were asked about their medical comorbidities, and they were dichotomized as follows: diabetes mellitus (have diabetes mellitus and no diabetes mellitus), hypertension (have hypertension and no hypertension), smoking status (never smokers and current smokers), chronic kidney disease (CKD) (have CKD and no CKD), and chronic obstructive pulmonary disease (COPD) (have COPD and no COPD). Body mass index (BMI) was measured during the survey data collection, and this was also dichotomized as obese versus not obese.

COVID-19 vaccination

Respondents were asked if they had been vaccinated against COVID-19, and they responded with a yes/no. Those who responded with a yes were then asked about the number of times they had been vaccinated against COVID-19. Their responses were one, two, three, and four or more times. This was dichotomized as <3 or ≥3 vaccinations. The three vaccines have been selected due to the seroconversion rates of COVID-19 vaccines, which are 39.4% before and 66.6% after the third dosage. The third dose is vital to protecting against all COVID-19 infections, risk of mortality, and severe disease.

Statistical analyses

Sampling weights (combined weighting) were applied to account for the survey's complex study design. The COVID-19 vaccination status of those who responded to the question "Ever been told you had coronary heart disease" was first established with weighted proportions reported. Furthermore, the number of vaccines received in the CHD group was determined, with a dichotomy created as those who received <3 vaccines and those who received three or more vaccines with weighted proportions reported. A Pearson's chi-square test was then used to determine group differences in COVID-19 vaccine status in respondents with CHD with counts and weighted proportions also reported. With the identified group differences, a logistic regression analysis was conducted to establish the predictors of COVID-19 in populations with CHD using the characteristics that differed as the independent variables. Stata 14.0 (Stata Corp LLC, College Station, TX) was used for statistical analysis, and p < 0.05 was set for a significance level in a two-tailed test.

## Results

**Figure 2:**
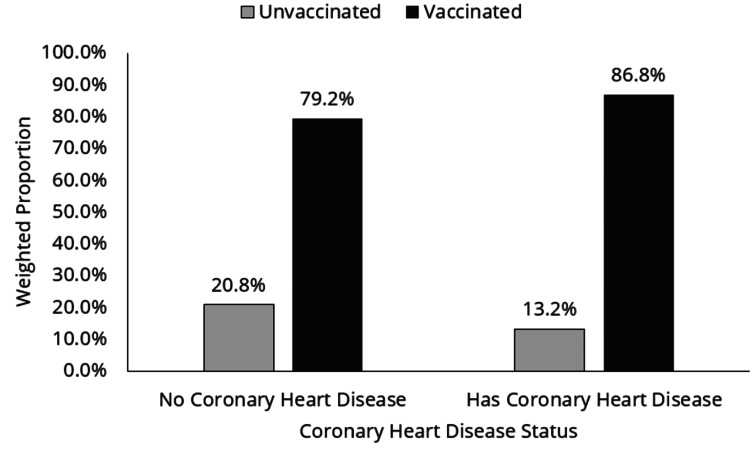
Distribution of CHD population by COVID-19 vaccine status CHD: coronary heart disease, COVID-19: coronavirus disease 2019

Figure 2 shows the distribution of respondents who answered yes or no to the question "Ever been told you had coronary heart disease" by COVID-19 vaccination status. Regardless of CHD status, a higher proportion of respondents reported being vaccinated against COVID-19 (p < 0.001); however, as compared to those without CHD, a larger proportion of those with CHD reported being vaccinated against COVID-19 (1,491 (86.6%) versus 2,100 (79.2%)).

**Figure 3:**
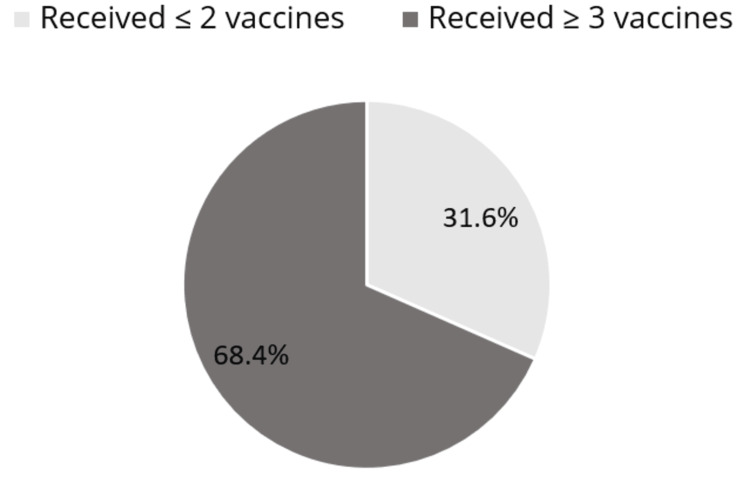
Distribution of the number of COVID-19 vaccines received by respondents with CHD CHD: coronary heart disease, COVID-19: coronavirus disease 2019

Figure 3 shows the distribution of respondents with CHD who have received ≤2 vaccines or three or more vaccines. Most of the population reported receiving three or more vaccines against COVID-19 (1,065 (68.4%) versus 425 (31.6%), p < 0.001).

Table 1 shows the weighted proportion and count of respondents with CHD who reported receiving the COVID-19 vaccine. Among the total population, the majority were 65 years of age and above, male, non-Hispanic white, insured, and did not have a college degree. As compared to those who reportedly had not been vaccinated, those who were vaccinated were older, age ≥ 65 years (1,158 (89.9%) versus 136 (10.1%), p < 0.001), had a ratio of family income to poverty threshold of 1 and above (1,312 (88.4%) versus 158 (11.6%), p < 0.001), did not have a college degree (1,018 (84.7%) versus 182 (15.3%), p < 0.001), and were less likely to report a history of COPD (1,227 (88.6%) versus 148 (11.4%), p < 0.001).

**Table 1:** COVID-19 vaccination status of respondents with coronary heart disease COVID-19: coronavirus disease 2019, NH: non-Hispanic, DM: diabetes mellitus, COPD: chronic obstructive pulmonary disease, -: intentionally left blank

Characteristics		Total (number (%))	No COVID-19 vaccine (number (%)) (217 (13.2%))	Received COVID-19 vaccine (number (%)) (1,491 (86.8%))	p
Age (years)	-	-	-	-	<0.001
	40-64	389 (100%)	76 (19%)	313 (81%)	
	65+	1,319 (100%)	141 (10.1%)	1,178 (89.9%)	
Sex	-	-	-	-	0.774
	Female	701 (100%)	97 (13.5%)	604 (86.5%)	
	Male	1,007 (100%)	120 (13%)	887 (87%)	
Ethnicity	-	-	-	-	0.146
	NH white	1,321 (100%)	171 (14.4%)	1,150 (85.6%)	
	Hispanic	128 (100%)	15 (10.1%)	3,112 (89.9%)	
	NH black	165 (100%)	20 (10.4%)	2,344 (89.6%)	
	NH others	94 (100%)	11 (7.9%)	2,079 (92.1%)	
Ratio of family income to poverty threshold	-	-	-	-	<0.001
	<1	238 (100%)	59 (23%)	179 (77%)	
	1+	1,470 (100%)	158 (11.6%)	1,312 (88.4%)	
College degree	-	-	-	-	<0.001
	No college	1,200 (100%)	182 (15.3%)	1,018 (84.7%)	
	College	496 (100%)	29 (5.3%)	467 (94.7%)	
Insurance	-	-	-	-	0.102
	Uninsured	30 (100%)	9 (24.6%)	21 (75.5%)	
	Insured	1,678 (100%)	208 (12.9%)	1,470 (87.1%)	
Obesity	-	-	-	-	0.842
	Not obese	1,053 (100%)	133 (13.1%)	920 (86.9%)	
	Obese	627 (100%)	82 (13.5%)	545 (86.5%)	
Smoking status	-	-	-	-	0.089
	Never	776 (100%)	82 (11.4%)	694 (88.6%)	
	Now/former	899 (100%)	130 (14.8%)	769 (85.2%)	
DM	-	-	-	-	0.129
	No DM	1,203 (100%)	143 (12.3%)	1,060 (87.7%)	
	DM	500 (100%)	73 (15.2%)	427 (84.8%)	
Hypertension	-	-	-	-	0.672
	No hypertension	370 (100%)	327 (87.8%)	14,000 (77.9%)	
	Have hypertension	1,338 (100%)	1,164 (86.6%)	8,480 (83.3%)	
COPD	-	-	-	-	<0.001
	No COPD	1,375 (100%)	148 (11.4%)	1,227 (88.6%)	
	Have COPD	326 (100%)	69 (21.5%)	257 (78.5%)	

Table 2 shows the predictors of COVID-19 vaccination in populations with coronary heart disease. Older age (odds ratio (OR): 2.01 (95% confidence interval (CI): 1.40-2.89), p < 0.001), ethnic minorities, a ratio of family income to poverty threshold of 1 and above (OR: 2.40 (95% CI: 1.58-3.64), p < 0.001), having a college degree (OR: 3.09 (95% CI: 1.85-5.14), p < 0.001), and being insured (OR: 3.26 (95% CI: 1.03-10.26), p = 0.044) were positive predictors of being vaccinated against COVID-19. Obesity, diabetes mellitus, hypertension, and smoking cigarettes were not statistically significant predictors of COVID-19 vaccination.

**Table 2:** Predictors of COVID-19 vaccination in populations with coronary heart disease COVID-19: coronavirus disease 2019, -: intentionally left blank, Ref: reference group, DM: diabetes mellitus, NH: non-Hispanic, CI: confidence interval

Characteristics	Odds ratio (95% CI)	P
Age (years)	-	-
40-64	Ref	-
65+	2.01 (1.40-2.89)	<0.001
Sex	-	-
Female	Ref	
Male	1.12 (0.80-1.59)	0.509
Ethnicity	-	-
NH white	Ref	-
Hispanic	2.28 (1.05-4.96)	0.038
NH black	2.53 (1.44-4.44)	0.010
NH others	2.63 (1.05-6.62)	0.040
Ratio of family income to poverty threshold	-	-
<1	Ref	
1+	2.40 (1.58-3.64)	<0.001
College degree	-	-
No college	Ref	-
College	3.09 (1.85-5.14)	<0.001
Insurance	-	-
Uninsured	Ref	
Insured	3.26 (1.03-10.26)	0.044
Obesity	-	-
Not obese	Ref	-
Obese	1.15 (0.78-1.69)	0.481
Smoking Status	-	-
Never	Ref	
Now/former	1.00 (0.64-1.42)	0.822
DM	-	-
No DM	Ref	-
DM	0.78 (0.55-1.10)	0.159
Hypertension	-	-
No hypertension	Ref	
Have hypertension	0.99 (0.58-1.72)	0.995

## Discussion

Among adults aged 40 and above with CHD, 86.8% reported COVID-19 vaccination, with 68.4% receiving more than two doses. Our findings underscore older age, ethnicity, and socioeconomic and educational disparities impacting COVID-19 vaccine uptake in this population. Specifically, older age showed a doubled likelihood of vaccination, while income above the poverty threshold tripled the odds. Possessing a college degree and having insurance were also strong indicators, with threefold and over threefold increased odds of vaccination, respectively. Interestingly, other established health conditions such as obesity, diabetes mellitus, hypertension, and smoking did not significantly predict vaccination uptake within this cohort.

We found an 86.8% vaccination rate among adults aged 40 and above with coronary heart disease against COVID-19, highlighting a high vaccination uptake within this vulnerable population. This rate is similar to the 92%-95% vaccination rate according to the Centers for Disease Control and Prevention (CDC) of the general US adult population who have received at least one dose of the COVID-19 vaccine [13]. In addition, in this study, more adults 65 years and above reported receiving the vaccine as compared to those 40-64 years of age and more men as compared to women, a finding consistent with that reported of the general population with regard to age but inconsistent with regard to sex [14]. This shows that although overall, 40+ adults with CHD have a high uptake of the COVID-19 vaccine, there are discrepancies suggesting a potential age-based disparity in vaccination rates within the heart disease population, warranting further investigation into age-related vaccination behaviors and access to healthcare resources.

Moreover, a study conducted by the CDC in 2022 showed a significantly lower vaccination rate among individuals with cardiovascular diseases compared to those without comorbidities, emphasizing disparities in vaccine uptake among specific health condition cohorts [15]. These divergent findings highlight the need for targeted interventions and outreach efforts to improve vaccination rates among individuals with heart disease across different age groups. Factors such as vaccine accessibility, awareness campaigns tailored to specific health conditions, and addressing hesitancy through targeted education could contribute to narrowing the vaccination gap among populations with coronary heart disease [13-16].

Our study indicating a 68.4% rate of adults aged 40+ with coronary heart disease receiving more than two COVID-19 vaccine doses reflects proactive vaccination efforts. Comparatively, a broader study across various health conditions found a lower rate of additional doses at 55%, suggesting potential disparities in booster uptake among different health cohorts [16]. While the 68.4% vaccine booster rate highlights commendable adherence within the heart disease group, strategies to enhance booster awareness and accessibility could further improve additional dose uptake, ensuring robust protection against emerging variants and sustained immunity in this vulnerable population.

Older age is a consistent predictor of COVID-19 vaccination. Several studies, including those focused on different health conditions or general populations, consistently identify older age as a significant predictor of COVID-19 vaccination [17-19], with younger age continuously associated with COVID-19 vaccine hesitancy [20]. The increased vulnerability of older individuals to severe COVID-19 outcomes likely motivates this higher vaccination uptake, while younger adults with fewer or no comorbidities who are less likely to experience severe COVID-19 outcomes along with fears about side effects are less motivated to get vaccinated.

With regard to socioeconomic factors, in this cohort of adults with coronary heart disease, socioeconomic factors such as income-to-poverty ratio, educational attainment, and insurance status significantly influence vaccination behavior. This aligns with broader studies indicating disparities in vaccination rates based on socioeconomic status [21,22]. Thus, a better socioeconomic status, which could be seen as equivalent to better health literacy, once again has proven to be a significant factor in the uptake of preventive measures in populations with CHD [23-25], in this case, vaccination against COVID-19.

In contrast with other populations, a study exploring predictors of vaccination often highlights the impact of health-related factors such as obesity, diabetes, hypertension, and smoking on vaccination decisions [26]. However, within the cohort of adults 40+ with coronary heart disease, these factors did not significantly predict vaccination uptake, findings consistent with a US study of the general population that showed that underlying medical conditions or morbid obesity did not influence vaccine decisions [27]. This finding suggests that medical comorbidities may not affect vaccine decisions as much as sociodemographic factors, thus buttressing the earlier points made about strategies to address sociodemographic gaps to boost vaccine awareness and uptake in populations with CHD.

Our studies have some strengths worthy of note. This study made use of a nationally representative sample of US adults with CHD, and sampling weights were applied in the analysis to provide national estimates. We evaluated the issue of COVID-19 vaccination uptake, a topic not formerly addressed in the literature, using the 2022 data from the CDC.

While the study provides valuable insights into the vaccination patterns among adults with coronary heart disease, it is important to acknowledge some limitations. Firstly, the study relies on self-reported data obtained from the National Health Interview Survey, which may introduce recall bias and inaccuracies, as individuals may misremember or selectively report their vaccination status. Since it is a nonexperimental study, causation cannot be implied, and as such, findings from this study should be applied with caution. Secondly, the study focuses solely on adults with coronary heart disease, limiting the generalizability of its findings to the broader population. This narrow scope may overlook nuances in vaccine uptake among other demographic groups, potentially missing important factors influencing vaccination behavior. Thirdly, sampling bias is another potential limitation acknowledged in the present study. The study findings are likely to be influenced by sampling bias, given that the NHIS and data might not effectively capture individuals and subgroups underrepresented in the survey, which may, in turn, impact the generalizability of the findings. Lastly, the study is based on data from 2022, and given the evolving nature of the COVID-19 pandemic and vaccination campaigns, the findings might not reflect the current landscape, emphasizing the need for ongoing research to capture the dynamic nature of vaccination trends.

## Conclusions

In conclusion, the findings of this study indicate that regardless of CHD status, 86.6% of the respondents reported being vaccinated against COVID-19 in comparison to 79.2% of respondents with CHD who reported being vaccinated against COVID-19. Of the study population, 68.4% reported receiving more than three vaccination doses against COVID-19. Further, the findings have indicated that older persons (65 years and above), at 89.9%, reported being vaccinated than younger persons (below 40 years). The key predictors of COVID-19 vaccination uptake in populations with coronary heart disease were found to be age, ethnic minority, family income level, insurance status, and education level. Thus, the findings of this study emphasize that understanding the unique predictors within the coronary heart disease population is critical for targeted interventions. While age remains a consistent factor, socioeconomic determinants such as income, education, and insurance status likely influence COVID-19 vaccination uptake. This necessitates the development of novel strategies for creating awareness of the importance of vaccination uptake among persons with CHD. Further, the study finding indicating that the traditional health-related variables impacting vaccination decisions in the general population might not be important in the CHD cohort necessitates further research aimed at understanding the complexities underlying vaccination uptake and hesitancy in the persons with CHD subgroup. Such studies will aid in enabling in-depth comprehension of the various complexities underlying vaccine uptake and the development of better interventions and strategies for increased vaccine uptake by persons with CHD. More research is also needed to improve public health strategies and ensure that this vulnerable population has fair access to vaccinations.
